# Dr. Paul Schoeppe

**Published:** 1869-12

**Authors:** 


### Miscellaneous.

### Dr. Paul Schoeppe.

We have received the following communication in reference to this most interesting case, and as we think it advisable to consider whatever has a bearing upon the question of Dr. Schoeppe's guilt or innocence before rather than after his execution, we lay it before our readers:

WASHINGTON, D. C., Dec. 8, 1869.

*To the Editor of the Chronicle:*

The case of the unfortunate Dr. Paul Schoeppe, who was convicted in June last, at Carlisle, Pa., of the alleged murder of Miss Steinnecke, a patient of his, on purely circumstantial and expert testimony, is exciting comment in all professional and educated circles throughout the country. We gather the following facts from what has been published from time to time regarding the case.

The Doctor is a young man, about twenty-five years of age, a German by birth, and a graduate of the University of Heidelburg. For some years back he has been practicing his profession with success and reputation at Carlisle, Pa., where his father resided and had charge of the Lutheran congregation.

During the summer of 1868 Miss Steinnecke, a maiden lady, resident at Baltimore, and full habit, about sixty-five years of age, visited Carlisle, where she had relatives. While there, owing to some indisposition, she consulted Dr. Schoeppe. The acquaintance thus begun proved agreeable, and was followed by a proffer of marriage, which was accepted. After the lady's return to Baltimore a correspondence was regularly carried on and business matters discussed. The confidence of each in the other was satisfactory and complete, the lady advancing money to enable the Doctor to extend his business interests. In the fall Miss S. returned to Carlisle, having previously requested, by letter, the Doctor to secure pleasant rooms for her at a hotel. She seemed to be in her usual health, but the correspondence exhibited on the trial shows she had been taking medicine, and was also under treatment by an oculist in Baltimore for some affection of the eyes. This fact and its probable significance as a symptom of Bright's disease, which, if the kidneys had been properly examined, might have been verified, seems to have been lost sight of on the trial.

Nothing disturbed the harmony of their friendship or intercourse until Miss S. was taken sick on the 27th of January, and dying on the 28th, 1869. She was attended by two physicians, Schoeppe and Hermon, the latter having been called in by the former as soon as he found the case assuming a serious aspect.

The only medicine given, that is in evidence, was an emetic, composed of two grains of tartar emetic and ten of ipecac, administered by Dr. Schoeppe, at the commencement of her illness, to relieve an oppression of the stomach from a supper of beefsteak eaten the day before. The medicine acted properly, but the patient became prostrated, perhaps paralyzed, followed by profound stupor, ending in death about thirty-four hours after the commencement of the attack.

Her remains were taken in charge by her relatives and removed to Baltimore for interment, accompanied by the Doctor as a mourner. On the 1st of February, the Doctor, through his counsel, filed in the Probate Court of Baltimore a document, purporting to be the last will and testament of Miss Maria Steinnecke, dated December 3, 1868, whereby all her property was left to Dr. Schoeppe, her lover and physician. The subscribing witnesses to the will are Dr. Schoeppe and F. Schoeppe, his father.

Up to this time there had not been the least suspicion in the mind of any one that there had been foul play, or that her death presented anything violent or unnatural. But, immediately upon the production of this document, which disposed of her property so contrary to the expectation of her natural heirs, that they at once pronounced it a forgery, and shortly after declared their suspicions that the Doctor had murdered the testator. The accusation was horrifying, and seemed to gather strength from its enormity and the constant prompting of her disappointed heirs. No pains have been taken to prove a forgery, and if a forgery, whether before or after the death of Miss Steinnecke.

At this point in the history of the case begin proceedings of the greatest interest to justice and humanity. On the 10th of February, thirteen days after death, the body of Miss S. was taken from the grave and subjected to a *post mortem* to find poison, as it was alleged she had died a violent death from the administration of prussic acid. A professor of chemistry of fair repute, at the fee of $250, was employed to make chemical tests of the contents of the stomach and intestines, for its discovery. Young men of no established reputation as pathologists were employed to make the autopsy. The evidence of these experts, as detailed before the coroner's jury, and on the trial, shows that their examinations were partial and inconclusive. As poison was suspected, almost all their inquiry was directed to its discovery. The chemist, from the mode in which he manipulated the contents of the stomach and intestines, failed, as signally as the pathologists, to deduce opinions of any value. His report would scarcely do credit to a first-class medical student, and the incompleteness of the autopsy would have prevented even any intelligent or profitable discussion of the case before an ordinary medical society. But, unfortunately, the opinions deduced from their hasty, incomplete, and unreliable examinations of the body and the contents of the stomach, laid the fatal foundation for all the subsequent proceedings and prejudices against the Doctor. There is not a pathologist or chemist, we believe, of any standing, in the whole country, that will pretend to sustain the deductions of either; yet, upon these opinions, (for they are unworthy of being called evidence,) the coroner's jury found a verdict of death from poison. The same testimony led to the Doctor's indictment, by the grand jury of Cumberland county, Pa., for "murder and manslaughter."

When arrested and imprisoned, public opinion became more and more inflamed against him; being a foreigner, without means and influential friends, he was forced to trial at great disadvantage, while the minds of the people were still excited. A singular, if not an erroneous proceeding in the case, was permitted by the court in allowing hypothetical questions as to the cause of death to be put by the prosecution to witnesses, which proceeding assumed as a basis that other than natural causes were present. Not one particle of positive evidence of any kind was given on the trial that poison had been given to the patient. The chemist thought he had found "faint traces of prussic acid;" but declared positively that no narcotic drug was present, nor was it shown that the symptoms present during the brief illness of Miss S. were such as could not be accounted for by natural causes in a person of her years and physique.

The trial developed most conclusively to all dispassionate readers of the report given of it that the prosecution failed utterly to prove the *corpus delicti*, and that the contrary clearly appeared that death ensued from natural causes; and further, it was particularly made evident that the facts relied upon by the prosecution and the means employed by the chemist to prove the presence of prussic acid in the stomach were utterly unreliable, and made the very poison he was in search of, the tests he used not being the most approved or exact. So complete was the error of the chemist made to appear by the evidence of other experts and competent chemists, that the judge, in charging the jury, felt called upon to direct them to reject the prussic acid theory and inquire into narcotics, which the testimony of Dr. Aikin, as we have just stated, was that he had looked or tested for all the ordinary mineral and vegetable poisons, including morphia and strychnine, but none were present. But that monster, suspicion, had firm hold upon public opinion, which had already adjudged the doctor guilty and were determined to punish him. The indictment against the Doctor charged him with wilfully taking the life of Miss Steinnecke, by the administration of poison; and, although not one iota of evidence from beginning to end went to sustain this allegation, yet the jury found him guilty of murder as indicated.

The judge is represented as somewhat surprised at the verdict, as were those who frequented the court room during the trial; but, nevertheless, as was perhaps his duty, he passed the sentence of the court in accordance with the verdict of the jury, who, in criminal cases in Pennsylvania, are judges of the law and the facts.

We know nothing of the ability of the Doctor's counsel, but infer from the published reports, that they exhibited no special ability in developing errors or false facts in the testimony of the witnesses of the prosecution, or in establishing the facts of the previous health of the deceased. We think it very probable that had the oculist, and perhaps other physicians, who had attended the deceased, and persons who were familiar with her daily habits, been summoned, it would have been made apparent that she was not as healthy a person as was generally supposed. According to the testimony of Wm. Kennedy, she made "startling noises in her sleep, as some one in great pain or distress," which was sufficient to awake him in an adjoining room at the hotel.

William Drew met Miss S. on the street the day before she died, to whom she complained "of feeling very bad, and of giddiness of the head."

Dr. Ridgly, a distant relative of the deceased, and who assisted at the *post mortem*, said that Miss Steinnecke was in the habit of "grunting," which he understood to mean complaining without a cause. No doubt other more direct and positive facts in this direction might have been adduced.

An arrest of judgment and a motion for a new trial was made, but refused, and the judge passed sentence. The case then could only go to the Supreme Court on a writ of error, as no review is granted in similar cases. Here the writ of error was not sustained, the technicalities of the record being found correct. The poor Doctor who had relied upon justice, and sustained his firmness thus far, was now reduced to despair. The publication of the trial, and the evidence upon which he had been convicted, however, had by this time attracted a good deal of attention among chemists and physicians. who plainly saw that the doctor was in danger of being sacrificed to the technicalities of the law. Numerous strong appeals have been made to Governor Geary by physicians, chemists, and medical societies from various States and large cities, all asserting their conviction that the guilt or the administration of poison by the doctor to Miss Steinnecke has not been proven. As to the allegation that the doctor has forged a check or a will, the medical profession know nothing of the facts, and are in no sense apologists of crime. If the fact is proven, let him suffer the full penalty of the law.

Governor Geary very naturally referred the questions raised and the appeals made for Dr. Schoeppe's pardon to his legal adviser, Attorney General C. F. Brewster, who had just been initiated into office. This officer makes a superficial, running synopsis of the case, scarcely touching the facts, which should prove conclusively to the Governor that conviction in this case was the result of excited feeling and the undue influence given to, as has been claimed, unreliable expert testimony. The Attorney General says, "the whole question was thoroughly discussed and fairly submitted to the tribunal selected for its solution—the jury of the vicinage. They have settled it, and with them rests the responsibility." He then flatters the court and the jury, and ends by advising the Governor to decline to review the questions passed upon by the court and the jury. Upon this the Governor affixed his signature to the death-warrant of Dr. Paul Schoeppe, to be executed on the 22d instant.

We cannot help, in view of all the facts, but feel that this is about to be a judicial murder, which the Governor's prerogative of pardon should avert.—*Washington Morning Chronicle.*

